# Supplementary material for: Effects of Short-Term Inhibition of Rho Kinase on Dromedary Camel Oocyte In Vitro Maturation
Source: Animals (Basel). 2020 Apr 25;10(5):750. doi: 10.3390/ani10050750 (PMC7277376; doi:10.3390/ani10050750)
Supplement: Supplementary file 1 [file animals-10-00750-s001.pdf]

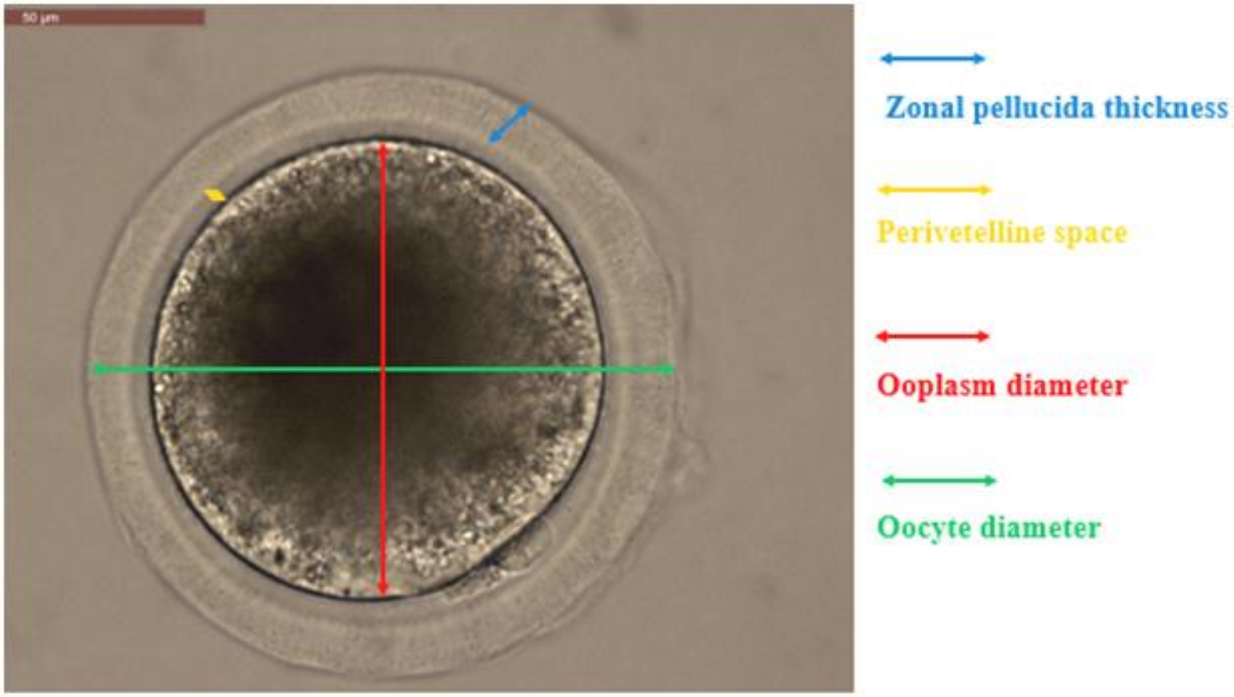

Figure S1: Camel oocyte morphometry parameters. Scale bar = 50 µm.

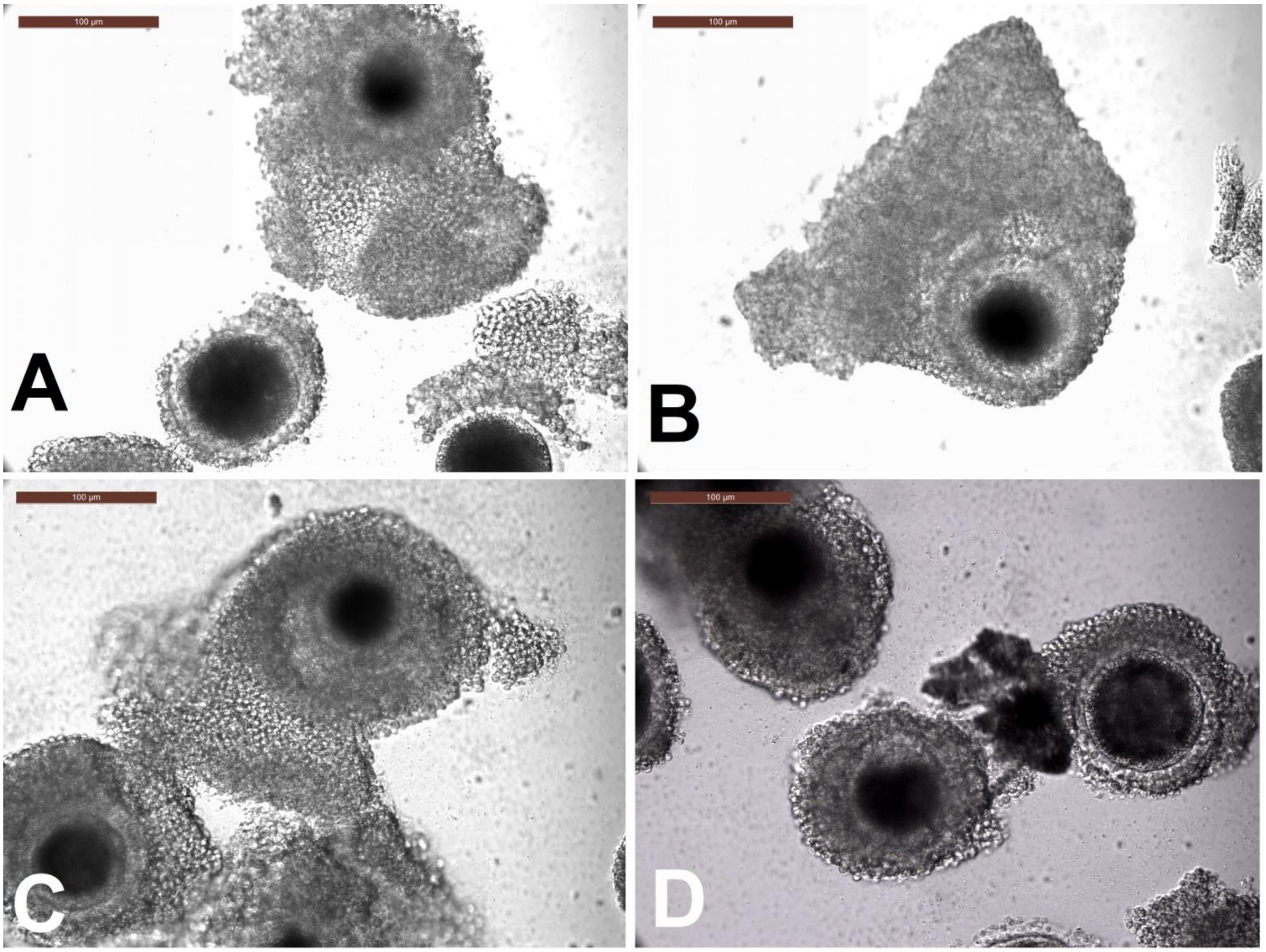

Figure S2: Effect of short-term supplementation on cumulus morphology of COCs. A: Control group, B: Cumulus morphology after pre-IVM with RI for 2 hour, C: Cumulus morphology after pre-IVM with RI for 4 hour, and D: Cumulus morphology after pre-IVM with RI for 6 hour. All groups were in comparison with control group and cumulus morphology after pre-IVM showed no significant difference. Scale bar = 100 µm.
